# Sustainable agriculture and GM crops: the case of *Bt* cotton impact in Ballari district of India

**DOI:** 10.3389/fpls.2023.1102395

**Published:** 2023-08-30

**Authors:** Arjunan Subramanian

**Affiliations:** Economics, Adam Smith Business School, University of Glasgow, Glasgow, United Kingdom

**Keywords:** sustainable agriculture, *Bt* cotton, pest pressure, genetically modified crops, India

## Abstract

Effects of *Bacillus thuringiensis* (*Bt*) cotton are at the forefront of an intense debate on the benefits of genetically modified (GM) crops among smallholder farmers in developing countries. Existing studies fail to control for confounders, selection bias, or cultivation bias from preferential treatment in the initial adoption phase. Addressing these concerns in this paper, I examine the impact of *Bt* cotton employing an unbalanced panel fixed-effects model of a crop yield and profit function on newly collected plot-level data in the most recent decade. Results show that *Bt* cotton yields have stagnated, have a null effect on profits, and have become more sensitive to pest pressure in the most recent decade. Though many studies have demonstrated higher crop yield and profit gains in the first decade of *Bt* cotton adoption that raised the average returns to the technology, the second decade shows convergence in benefits, which raises obvious questions about the prospect of GM technology. Since *Bt* cotton is the only GM crop technology widely adopted by smallholder farmers, the findings of this paper contribute to the broader public debate on the future of agricultural biotechnology.

## 1 Introduction

World food requirements will likely increase with dietary habits, such as higher meat and dairy consumption from rising per capita incomes. The pressure on global agriculture will increase, with demand for crops expected to double by 2050 roughly (Godfray et al., 2010; Tilman et al., 2011; United Nations, 2022). Even as we face these future burdens, there have been renewed investments in novel technologies to boost productivity. One of the most salient developments in global agriculture is the introduction of Genetically Modified (GM) crops (Aziz et al., 2022). GM crops, though, have been commercially adopted in both developed and developing countries over the past two decades, a fierce debate continues to rage concerning its implications for smallholder farmers in developing countries (Barrows et al., 2014; Abedullah et al., 2015; Bakhsh, 2017; Tokel et al., 2021; Evanega et al., 2022).

At the centre of this debate are the socioeconomic effects of *Bacillus thuringiensis* (*Bt*) cotton (Tabashnik et al., 2013; Zilberman et al., 2018; Smyth, 2020). The short-term benefits of *Bt* cotton are well documented, but the long-run impact is subject to substantial debate. Recent studies from China show positive benefits of *Bt* cotton in both the short-run and long-run, using farm-level and aggregate provincial-level data (Qiao, 2015; Qiao and Huang, 2020). Using farm-level data from India and Pakistan, studies have shown the short-term benefit (Subramanian and Qaim, 2009; Subramanian and Qaim, 2010; Subramanian et al., 2010; Bakhsh, 2017), but the long-term impact with aggregate data shows no gains for India (Kranthi and Stone, 2020). The debate about the sustainability of the *Bt* cotton gains is still a hot topic in India; thus, in this paper, I focus on India.

Even though farm-level studies have shown sizable gains in the first decade of *Bt* cotton adoption, these studies control for variables causing spurious associations affecting *Bt* adoption and yield growth (confounders) and selection bias from early *Bt* adopters being an unrepresentative group of progressive farmers. However, they suffer from cultivation bias due to preferential treatment for the costly *Bt* seeds in the initial adoption phase (Kranthi and Stone, 2020).

On the other hand, studies using aggregate data over two decades at the State- or National-level show mixed results (Plewis, 2019; Kranthi and Stone, 2020). These studies either do not distinguish late adopters from early adopters and non-adopters, thus suffering from selection bias or the analysis of aggregate data masks the effect size’s spatial heterogeneity. The benefits of *Bt* cotton can also change over time from pest pressure and resistance development, the effectiveness of sprays, availability of substitutes, and other dynamics (Kranthi and Stone, 2020; Lu et al., 2022). Yet, farm-level primary data studies addressing how individual farmers responded to the recent decade of *Bt* cotton adoption remain scarce.

From a policy perspective, it is interesting to examine the performance of *Bt* cotton, which has serious implications for the acceptance, regulatory approval, and adoption of GM food crops. For instance, the Chinese government has delayed commercialising many GM crops, including GM rice (Jin et al., 2019). Similarly, India has also put on hold the approval for three food crops, *Bt* mustard, potato and eggplant, pending further evidence on the impact of *Bt* cotton (Ramaswami and Pray, 2005; Jayaraman, 2010; Herring, 2015). Unfortunately, data nonavailability in India prevents a plot-level analysis of cross-state patterns that can address the above concerns.

This paper fills the gap using the newly collected detailed plot-level panel household data over five years in the recent decade from cotton farmers in the Ballari district of the Indian State of Karnataka. Because of the universal adoption of *Bt* cotton in the second decade of adoption, the data do not have non-*Bt* cotton plots. Though I cannot generalise the results, I dig deeper, applying trend and regression analysis to the performance of *Bt* cotton yields and profits. Since the farm-level data comes from the second decade of the *Bt* cotton adoption (Subramanian, 2018), I provide, unlike previous studies, new micro-level evidence controlling for confounders and addressing both selection bias and cultivation bias. Because of the near-universal adoption of *Bt* cotton in the study region in the second decade, we can dismiss the concerns arising from the differential adoption rate of *Bt* technology resulting in the selection bias.

## 2 Materials and methods

To address the shortcomings of existing studies, I use comprehensive micro-level panel data collected from India’s second decade, 2012 to 2017, of *Bt* cotton adoption (Subramanian, 2018). The ethics committee at the Indian Institute of Management in Bangalore, India, approved the protocols related to the study. All methods were carried out following relevant guidelines and regulations. I obtained written informed consent from all the study participants. After obtaining ethical approval, I surveyed cotton-growing farmers in the Ballari district in Karnataka, a southwestern state of India. From the Bhoomi database, a census of land ownership in Karnataka, I randomly sampled 320 households. I followed a two-stage procedure. In the first stage, I identified all the villages predominantly growing cotton and randomly selected some households across these villages in the second stage.

I conducted four farm surveys among Indian cotton farmers between 2012 and 2017. A clustered random sampling procedure was followed to enlist the farmers from the Ballari district for the study. The first wave was implemented in March 2013, covering the 2012-2013 agricultural year. The second follow-up wave was implemented the following year, but the third and fourth waves were conducted consecutively after one gap year. The attrition is low except for the final year of the survey when some households disadopted cotton cultivation.

The dataset is an unbalanced panel of crop plots of varying plot sizes over four years. The samples selected are at the household level, and data collected is at the plot level though each sample household cultivated at least one cotton plot. However, a few households cultivated more than one plot. Thus, the number of cotton plots is higher than that of households. The estimation strategy is not at the household level but disaggregated by crop plots. The trained enumerators visited the sampled households at home and on the farm to administer the survey.

The farm survey, which includes a production module, collected retrospectively detailed plot level information on crop cultivation, such as outputs and inputs used. I collected detailed information on the number of family and hired labour used in the cost module, their days and hours worked, input quantity and prices, and transport costs. I recorded this information for each crop and every farming operation. There are 33 other crops grown that include cereals, pulses, and vegetable crops, including paddy, bengal gram, horse gram, maize, red gram, sugarcane, sunflower, cowpea, barley, groundnut, castor, green gram, and a combination of several crops raised together. Other information collected includes farmer-specific characteristics and household structures.

The attrition is low except for the final year of the survey when households disadopted cotton cultivation. Since our study has unbalanced panel data over four years, the fixed effects (FE) and random effects (RE) methods are used for estimating the impact of *Bt* cotton on crop yield and profits. I report the Hausman test to assess whether the FE model is the appropriate model for our data. As suggested by Wooldridge (2010) pooled ordinary least squares (OLS) method is employed when different samples are selected each year, thus not appropriate for our data. Though I control many confounding factors, the estimates are not causal, as are other studies in this debate. Addressing causality is empirically challenging when adoption is not wholly exogenous and evolves with the gains from the technology.

I estimate the following specification:


$$O_{it}=\;\alpha_{0}+\beta_{1}Sh_{it}+\eta_{1}X_{it}+Y_{t}\;+\delta_{v}+\epsilon_{it}$$

$O_{it}$ is the outcome of interest (yield per acre, profit per acre) for household i in period t; $Sh_{it}$ is the share of *Bt* cotton area in total area cultivated by household i in time t; $X_{it}$ is the plot and household level control variables, $Y_{t}$ is year fixed effects, $\delta_{v}$ is group fixed effects, and $\epsilon_{it}$ is an error term. The effect of interest $\left(\beta_{1}\right)$ captures the average adoption impact of *Bt* technology on cotton yield and profits. A significant, positive value of $\beta_{1}$ indicates that yield and profits increase with the *Bt* cotton area.

The plot and household level controls include seed rate and the number of seeds in grams used per acre. The timing of sowing and harvest date in months, and the number of times the cotton plot was irrigated. I also include square terms as the control variables in the regressions to allow for nonlinear linkages between *Bt* cotton and input prices and quantity. I have pesticide quantity as a separate control variable to reflect infestation from sucking and chewing pests. Additional controls include age and education of the farmer in years, land owned, cultivated area and area under sharecropping in acres. The standard errors are clustered by household. I checked for heteroskedasticity in the data using scatter plots that do not show variations in outcome variables are more significant among large land size holding. Farmers who cultivated cotton had one or two equal size land plots. All the data analyses were conducted in Stata 17 (StataCorp).

## 3 Results

### 3.1 Summary statistics

The means and standard deviations (in parentheses) and additional variables are presented in **Table 1**. For a simple comparison, the first two columns are reproduced from another study based on data from four Southern and Central Indian States (Kathage and Qaim, 2012). Comparisons of means across columns 1-2 with columns 3-6 show that the means from the survey data collected for this paper are along the expected lines. For instance, pesticide cost and fertiliser rate per acre presented in columns 1 and 2 are reasonably comparable to the closest year, shown in Column 3. Thus, the survey data used in this paper somewhat represents India’s Southern and Central cotton-growing regions.

**Table 1:** Summary statistics.

Plot level information	2002-2004	2006-2008	2012-2013	2013-2014	2015-2016	2016-2017
	(1)	(2)	(3)	(4)	(5)	(6)
Seed cost (1,000 Rs/acre)	1.60 (0.43)	0.91 (0.32)	1.038 (0.225)	0.954 (0.186)	0.939 (0.103)	0.934 (0.284)
Seed rate (g/acre)	490.72 (114.23)	570.75 (160.93)	1163.262 (435.553)	1069.781 (488.353)	924.001 (410.335)	1025.701 (267.135)
Pesticide cost (1,000 Rs/acre)	1.43 (1.57)	1.07 (1.38)	1.278 (1.101)	1.879 (1.318)	1.564 (1.109)	1.518 (1.327)
Fertiliser (t/acre)	0.26 (0.16)	0.25 (0.15)	0.279 (0.191)	0.463 (0.298)	0.403 (0.283)	0.310 (0.139)
Fertiliser cost (1,000 Rs/acre)	n.a	n.a	3.811 (2.732)	6.754 (4.720)	6.258 (4.667)	4.453 (2.096)
Micronutrients (1,000 Rs/acre)	n.a	n.a	0.035 (0.132)	0.066 (0.166)	0.184 (0.295)	0.146 (0.117)
Yield (kg/acre)	705.40 (360.41)	829.03 (341.08)	657.321 (430.693)	975.551 (488.202)	380.342 (219.701)	654.627 (228.533)
Profit (1,000 Rs/acre)	6.14 (6.89)	10.32 (7.73)	-10.639 (44.162)	4.145 (31.591)	-5.248 (11.964)	18.178 (10.162)
Production cost (1,000 Rs/acre)	7.65 (2.94)	9.03 (5.12)	37.027 (46.891)	38.758 (28.098)	21.741 (9.381)	16.082 (5.545)
Revenue (1,000 Rs/acre)	13.79 (7.32)	19.35 (8.42)	26.388 (17.924)	42.904 (23.391)	16.493 (9.519)	34.261 (11.832)
Crop area (acres)	6.20 (6.73)	5.79 (4.60)	6.138 (5.159)	6.984 (7.687)	7.957 (9.731)	8.214 (8.872)
Cotton price (Rs/kg)	19.52 (2.69)	23.31 (4.05)	21.452 (13.921)	25.401 (16.772)	32.284 (26.872)	39.173 (32.312)
Share of cotton area in total cultivated area	n.a	n.a	0.680 (0.286)	0.681 (0.284)	0.678 (0.313)	0.718 (0.309)

Columns (1) and (2) are taken from Kathage and Qaim (2012). Mean values are shown with standard deviations in parentheses. “n.a” in columns (1) and (2) refers to “not available” because Kathage and Qaim (2012) do not report this in their paper. One acre is equal to 0.405 hectares.

### 3.2 Trend analysis

**Figure 1:**
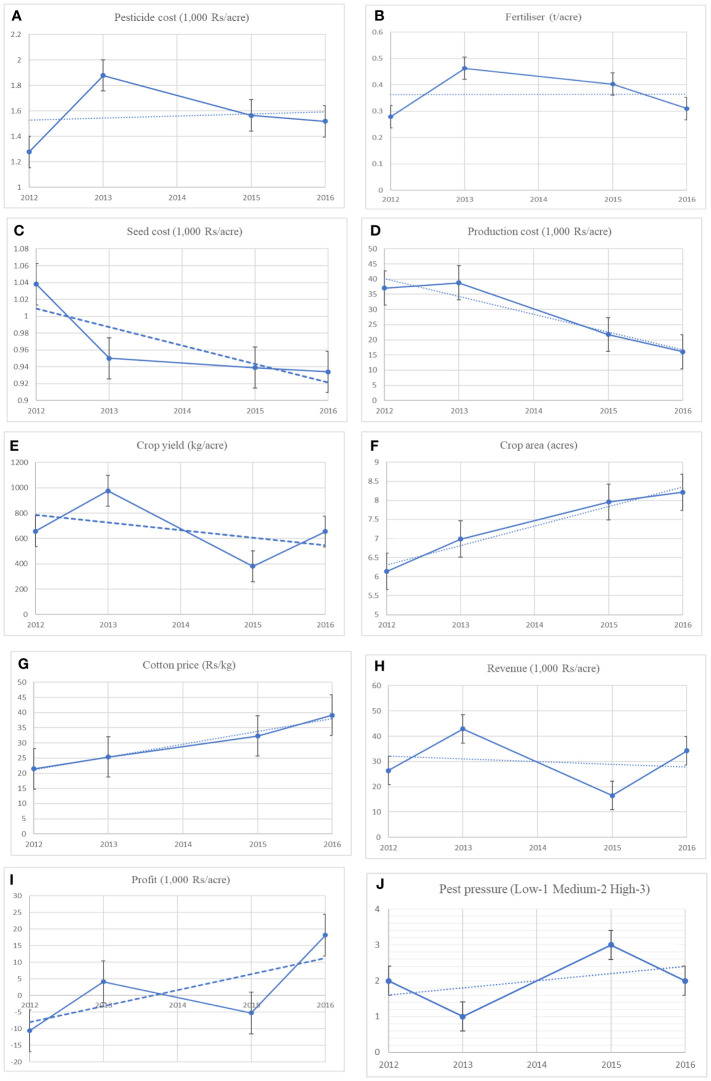
Trends in plot level household variables. Dashed lines are fitted trend lines over the years from 2002 to 2017. Cotton prices are for seed cotton farmers sell in the market. The graphs are plotted over the yearly mean from the plot-level data. The error bars are autogenerated for each variable. Fertiliser includes organic (manure and compost) and inorganic fertilizers (nitrogen, phosphorous, and potassium). All the cost figures are adjusted for inflation.

**Figure 1** shows the trends of some of the important variables in the *Bt* cotton controversy. The *Bt* cotton seed costs per acre changed very little over the years after the initial drop, with government policy limiting the maximum sale price of seeds. Despite increasing pest pressure, pesticide costs do not show an increasing trend. Though Gutierrez et al. (2019) suggest that pesticide use began to increase after 2012, the increase in pesticide cost, as shown in **Figure 1A**, was for just one year (2012-2013). A similar trend can also be observed in pesticide use for All-India in Figure 1a of Gutierrez et al. (2019). The decreasing trend in production cost (**Figure 1D**) may primarily reflect the decreasing trend in seed (**Figure 1C**) and pesticide costs and fertiliser use (**Figure 1B**). Note that production cost includes the labour cost of family and hired labour.

The cotton yields (**Figure 1E**) show a decreasing trend, although changing drastically between the years, with a somewhat steady increase in the share of the *Bt* cotton area (**Figure 1F**). The fertiliser use trajectory shows that an increased use improves crop yield while a decrease reduces it, echoing previous evidence (Gutierrez et al., 2019). Profits, which I calculate as the difference between revenue and cost of cultivation, appear to closely track the yields, turning positive when the yields are higher. However, given the falling trend for the cost of cultivation and a slight decrease in revenue, (**Figure 1H**) the profits reflect an increasing trend (**Figure 1I**).

Pest severity at the farm level was measured using a three-point scale: (1) level 1, low infestation; (2) level 2, moderate infestation; and (3) level 3, high infestation. Both yield and profits are highly sensitive to pest pressure reflected by the yearly fluxes (**Figure 1J**). Pest infestation problems can be severe due to adverse weather conditions in some years. Mostly pink bollworm infestation is highly related to rainfall and high level of humidity. When pest pressure is lower, yields and profits are higher. The *Bt* trait provided reasonable initial control of pink and American bollworms, but farmers were also tackling with insecticides secondary pests such as whitefly, jassids, mealybug, and aphids. By targeting lepidopteran pests, Bt cotton help improve management in many cotton-producing countries. In India, the pink bollworm evolved resistance to first and second generations of Bt cotton. Many recent studies have shown the return of the pink bollworm to the states in the central and southern zone of cotton production, which includes Gujarat, Madhya Pradesh, Maharashtra, Andhra Pradesh, Telangana, and Karnataka (Naik et al., 2018). Though second-generation *Bt* cotton containing Cry1Ac and Cry2Ab has replaced first-generation (Cry1Ac), several potential causes of the pest’s reoccurrence are currently debated (Najork et al., 2021).

The survey did not specifically distinguish between primary and secondary pests regarding the purpose of each insecticide sprayed. My understanding from the field is that farmers also targeted a few secondary pests, such as whitefly and aphids not controlled by the *Bt* trait. In 2015, the pink bollworm infestation was so high (**Figure 1J**) that farmers lost hope and thus stopped spraying insecticides. The lower pesticide cost for the same year, as in **Figure 1A**, is not from lower pest pressure but because farmers stopped spraying, fearing enormous irreversible losses from pests. The cotton yields dropped drastically (**Figure 1E**), resulting in lower revenues (**Figure 1H**) and, thus, lower profits (**Figure 1I**).

The decreasing trend in output is somewhat compensated by the increase in cotton prices (**Figure 1G**) to limit the decreasing trend in revenue. The above results show that farmers still benefit (profit) from *Bt* cotton adoption; however, it remains to be seen if these gains still hold after controlling for the confounding factors.

### 3.3 Regression analysis

In this section, controlling for the confounding variables, I examine the impact of *Bt* cotton using regression analysis with a standard fixed-effect panel data specification to address potential selection bias from attrition in adopting *Bt* cotton. I used unbalanced panel data over all four waves for the analysis. The Hausman test reported in **Tables 2**, **3** suggests that the FE model is the preferred model over the RE model. Results presented in **Table 2** report the effect of *Bt* cotton adoption on crop yield. Unlike many past studies that use dummy variables to indicate *Bt* cotton adoption, I compute the share of area under *Bt* cotton cultivation to the total cultivated area, hereafter referred to as the share variable. Since farmers also cultivate several crops apart from cotton, the share variable captures the dynamics in land use across farms. There are two advantages of using the share variable compared to a dummy: (a) dummy captures the substitution of land between *Bt* cotton and conventional cotton, ignoring other types of land-use change. (b) With the universal disadoption of conventional cotton, the *Bt* cotton adoption dummy loses its significance as a treatment variable.

**Table 2:** Impact of *Bt* cotton adoption on yield – fixed-effect model.

Plot level information	All-year panel		Two-year panel	
		2012-2013 & 2013-2014	2013-2014 & 2015-2016	2015-2016 & 2016-2017
	Coefficient (kg/acre)	Coefficient (kg/acre)	Coefficient (kg/acre)	Coefficient (kg/acre)
	(1)	(2)	(3)	(4)
*Bt* cotton area share	85.881 (139.173)	66.359 (297.050)	-601.960** (196.932)	752.294**(253.592)
Seed rate (gram/acre)	0.145* (0.077)	0.146 (0.117)	-0.758** (0.202)	0.822*** (0.193)
Sowing date (month)	-57.099 (51.114)	-37.370 (90.339)	-163.868 (224.379)	-101.114 (98.979)
Harvest date (month)	-3.243 (6.645)	44.082 (127.339)	-34.607** (10.081)	-14.842 (8.929)
Irrigation (number)	3.990 (6.109)	12.736 (12.439)	-27.060*(11.401)	5.272(7.304)
Price of seed bag (Rs/450g)	0.090 (0.158)	0.248 (0.242)	-1.130 (0.567)	0.757 (.605)
Fertiliser price (Rs/kg)	-0.259 (0.509)	-0.706 (0.895)	-2.440**(0.849)	2.582**(1.280)
Square of fertiliser price	-0.000 (0.000)	0.000(0.000)	0.001*(0.000)	-0.001*(0.000)
Pesticide price (Rs/lit)	0.135 (0.145)	-0.186 (0.282)	1.155**(0.300)	-0.235 (0.651)
Square of pesticide price	-0.000 (0.000)	0.000 (0.000)	-0.000** (0.000)	0.000 (0.000)
Pesticide per acre (lit)	-0.255 (0.114)	-0.330 (0.345)	-0.171 (0.204)	-0.180 (0.155)
Female wage rate (Rs/day)	-1.845* (1.084)	-4.004 (2.940)	3.164 (1.952)	-0.225 (2.543)
Square of female wage rate	0.010**(0.003)	0.013 (0.008)	-0.015 (0.013)	0.007 (0.009)
Micronutrient price (Rs/kg)	0.9538* (0.562)	-1.293 (1.892)	-3.828* (1.777)	-0.095 (0.650)
Square of micronutrient price	-0.002**(0.001)	0.001(0.005)	0.004 (0.003)	-0.000(0.001)
Manure use (ton)	-0.001(0.022)	0.035(0.039)	0.066 (0.040)	0.068 (0.061)
Square of manure use	-2.52e-07 (1.18e-06)	-7.33e-07 (1.90e-06)	-1.31e-06 (3.13e-06)	-0.000 (9.78e-06)
Cultivated area (acre)	-14.004**(7.249)	-7.753 (21.976)	-7.846 (12.637)	61.978**(25.271)
Area under sharecropping (acre)	-0.495 (18.944)	13.372 (35.970)	16.079 (42.943)	-576.661***(109.293)
Land owned (acre)	14.584*(8.101)	16.772 (21.797)	-0.949 (15.470)	-110.667**(54.323)
Age of farmer (year)	3.030(15.382)	454.758 (128.774)	-61.001** (17.780)	60.419 (68.676)
Education of farmer (year)	-16.342(22.408)		26.362 (19.675)	419.954***(85.632)
R-squared	0.691	0.403	0.347	0.506
No. of observation	1,254	688	667	566
Hausman test	30.05***	28.63**	68.04***	58.40***

The regressions also include year and village dummies and a constant term. *, **, *** significant at the 10%, 5% and 1% level, respectively. Fixed-effect models are estimated using household panel data with plot-wise information. Coefficient estimates are reported with standard errors in parentheses. In column 2, the variable Education of farmer was dropped due to collinearity. Cultivated area, the area under sharecropping (acre), land owned (acre), and age and education of farmers are at the household level required to control for confounders. The number of observations in columns 2 and 3 is higher than the number of households because a few households cultivated more than one cotton plot. The number of observations decreased in column 4 due to sample attrition in the final year.

**Table 3:** Impact of *Bt* cotton adoption on profit per acre – fixed-effect model.

Plot level information	All-year panel	Two-year panel		
		2012-2013 & 2013-2014	2013-2014 & 2015-2016	2015-2016 & 2016-2017
	Coefficient (Rs/acre)	Coefficient (Rs/acre)	Coefficient (Rs/acre)	Coefficient (Rs/acre)
	(1)	(2)	(3)	(4)
*Bt* cotton area share	12797.900 (8380.108)	-2776.609 (22025.39)	-28094.57**(11329.86)	37172.52**(13687.61)
Seed rate (gram/acre)	4.618 (4.655)	11.475 (8.732)	-17.580 (11.675)	26.577**(10.463)
Sowing date (month)	-1185.855 (3077.794)	4206.47 (6698.417)	-7126.984 (12908.95)	-5872.907 (5342.381)
Harvest date (month)	936.198**(400.133)	5261.881 (9441.839)	-2440.441***(580.014)	-322.447 (481.977)
Irrigation (number)	178.722 (367.882)	1253.448 (922.330)	-1125.817 (655.923)	182.335 (394.248)
Price of seed bag (Rs/450gram)	-13.505 (9.542)	-11.978 (17.944)	-35.308 (32.636)	17.462 (32.699)
Fertiliser price (Rs/kilogram)	45.081 (30.695)	69.249 (66.400)	-146.973**(48.895)	148.271**(69.107)
Square of fertiliser price	-0.032 (0.020)	-0.041 (0.043)	0.090**(0.032)	-0.093**(0.045)
Pesticide price (Rs/liter)	-6.481 (8.762)	-26.465 (20.947)	9.102 (17.2966)	-54.856 (35.164)
Square of pesticide price	0.002 (0.002)	0.007 (0.005)	-0.001 (0.006)	0.028 (0.021)
Pesticide per acre (liter)	-11.480 (6.922)	-51.430**(25.631)	-8.146 (11.773)	-10.295 (8.416)
Female wage rate (Rs/day)	42.182 (65.310)	-46.083 (218.009)	87.664 (112.354)	-11.458 (137.277)
Square of female wage rate	-0.311 (0.206)	-0.292 (0.650)	-0.570 (0.765)	0.311 (0.517)
Micronutrient price (Rs/kilogram)	55.716 (33.847)	-15.689 (140.298)	-285.313**(102.240)	42.720 (35.093)
Square of micronutrient price	-0.136**(0.064)	-0.144 (0.398)	0.461**(0.185)	-0.110*(0.063)
Manure use (ton)	-0.052 (1.343)	1.370 (2.899)	6.034**(2.342)	1.649 (3.343)
Square of manure use	-0.000 (0.000)	-0.000 (0.000)	-0.000 (0.000)	-0.000 (0.000)
Cultivated area (acre)	-384.175 (436.544)	-319.947 (1629.467)	509.354 (727.064)	2756.644**(1364.02)
Area under sharecropping (acre)	-539.862 (1140.7250	694.364 (2667.115)	4947.921 (2470.593)	-21584.16***(5899.076)
Land owned (acre)	808.373 (487.827)	1398.635 (1616.24)	-720.121 (890.057)	-2637.699 (2932.12)
Age of farmer (year)	857.984 (926.234)	23132.05*** (9548.241)	-1883.536 (1022.958)	5668.488 (3706.809)
Education of farmer (year)	471.408 (1349.301)	–	2985.999**(1131.95)	16304.12*** (4621.987)
R-squared	0.410	0.367	0.172	0.168
No. of observation	1,254	688	667	566
Hausman test	30.16**	22.05	40.41***	52.85***

The regressions also include year and village dummies and a constant term. *, **, *** significant at the 10%, 5% and 1% level, respectively. Fixed-effect models are estimated using household panel data with plot-wise information. Coefficient estimates are reported with standard errors in parentheses. In column 2, variable Education of farmer was dropped due to collinearity. The number of observations in columns 2 and 3 is higher than the number of households because a few households cultivated more than one cotton plot. The number of observations decreased in column 4 due to sample attrition in the final year.

Considering **Table 2**, in column (1), I estimate the panel fixed-effects model of a crop yield function combining data from all four years. I form two-year rolling panels in the rest of the columns to compare the yields sequentially with the previous survey year. Controlling for many confounders, increasing the share of the *Bt* cotton area did not significantly increase cotton output per acre, unlike what was observed in the first decade of *Bt* cotton adoption. The estimates align with previously noted trends of yield stagnation (Gutierrez et al., 2019; Kranthi and Stone, 2020). However, in the final year (column 4), the yield increased by 752 kg per acre (significant at 5%). Though many control variables are not statistically significant, I still keep these variables to confirm with the existing studies using similar models. However, I tried different variants of the current model, but the results did not change drastically for the *Bt* cotton area share variable.

In **Table 3**, I report results from estimating a fixed-effects specification of a profit function. The coefficient in column (1) shows that *Bt* cotton cultivation is not at all profitable. This average impact over the four-year period can mask the gains made in some years. Thus, I estimate using two-year panels to examine the annual effect of *Bt* cotton adoption. The results from the first year presented in column 2 show a negative impact, although not statistically significant. In the following year, the profits significantly decreased. In the final year in column (3), profits from *Bt* cotton plots increased by Rs. 37,172 (453 US$) per acre despite including all the control variables as in columns (2) and (3). Most notable is the significant increase in cultivated area. This result can be explained by both increases in yields and reductions in the cost of cultivation (See **Table 1**). The sharp rise in cotton prices (20%) and yield improved the revenue from cotton production. The fertiliser use fell by 29%, reducing production costs.

Since the sample size in the final year dropped by 15%, there is the possibility of attrition bias, where farmers who obtained lower-than-average yields dropped out of the sample. This dropout by the inefficient farmers could have potentially increased the profits in the final year. The analysis with different sub-samples, excluding the dropped-out farmers in the previous year, did not result in higher yields or profits. If these inefficient farmers are drawing down the profits, removing them from the sample should increase the outcomes. Thus, it is unlikely that attrition bias is the primary reason for improved profits in the final year.

## 4 Discussion

The cotton crop is grown in the subtropical and seasonally dry tropical areas in the northern and southern hemispheres. According to the International Cotton Advisory Committee, the leading producing countries in 2021-22 are India (25%), China (25%), the United States (16%), Brazil (12%), and Pakistan (5%). Cotton has been an economically important commercial crop for India since the earliest times and grows all four cultivated cotton species (Blaise and Kranthi, 2020). In 2000, *Gossypium hirsutum* represented 69% of the total cotton in India, followed by *G. arboreum* (17%), *G. herbaceum* (11%), and *G.barbadense* (3%). India has pioneered the hybrid cotton technology and has become the only country where most of its acreage is under hybrids. The hybrid technology prevents seed saving and requires annual purchases of high-cost seed that leads to sub-optimal planting densities (Gutierrez, 2018). After the introduction of *Bt* hybrids for commercial cultivation in 2002-03, the composition of cultivation of species drastically changed. Presently, all the cotton in India is under the *hirsutum* group (>95%, 2012), leaving only less than 5% under *arboretum* and *herbaceum* (Government of India, 2017).

GM technology provides novel methods and capabilities to enhance agricultural productivity, mitigate its environmental footprint, and sustainably feed growing populations (Zilberman et al., 2018). Though *Bt* cotton is not a yield enhancing technology, it is designed to protect the yield potential of the variety that carries the trait from damage from some pests (Gutierrez et al., 2015). Yet, several controversies surround its impact, posing barriers to broader adoption and diffusion (Aziz et al., 2022). We can distinguish three sets of studies. (1) Short-term studies are primarily based on farm-level data. (2) Aggregate (provincial- or state-level) data showing long-term impact. (3) Long-run effect using farm-level data. Though the short-term studies show sizeable gains, these benefits can be offset by increased pesticide use and secondary pest outbreaks, thus raising doubts about the sustainability of the benefits of *Bt* cotton. Given the initial adoption phase of the technology, these studies suffer from selection and cultivation bias.

Most studies from Pakistan are based on cross-sectional single-year data suffering from self-selection and endogeneity issues. An exception to these studies is Bakhsh (2017) and Bakhsh et al. (2016), which use panel data for 2008 and 2009, though still in the initial phase of *Bt* cotton, show yields far less than previous studies. Though *Bt* cotton varieties in Pakistan were already under cultivation, it was only approved by the National Biosafety Committee in 2010, with further approvals in 2014 (Bakhsh et al., 2016).

Studies using aggregate data such as national and sub-national levels are divided on the economic gains from *Bt* cotton. Recent studies from China show increasing long-run economic benefits using both aggregate provincial-level data and farm-level panel survey data. However, studies based on aggregated secondary data from India over extended periods show modest benefits of *Bt* technology. Other studies show higher fertiliser use, better irrigation facility, and farmers’ bias towards the supply of other inputs to *Bt* relative to non-*Bt* cotton are the primary reason for the gains in cotton yields (Gutierrez et al., 2019).

Our results from the second decade using farm level panel data show yield sensitivity to pest pressure has increased, resulting in losses in some years and overall stagnation. Similar recent reports of pest attacks have been documented in other states, such as Gujarat, Madhya Pradesh, Maharashtra, Andhra Pradesh, and Telangana (Najork et al., 2021). Studies have demonstrated the significance of weather as an essential driver in heightening pest outbreak risks (Gutierrez et al., 2015; Zhang et al., 2018). These studies highlight changes in land use, climate and agricultural technologies that affect pest severity and management. The mirid bugs are likely to increase in severity with warmer temperatures and reduced insecticide spraying against bollworms. Climate variability is expected to underscore the challenges of meeting increasing global agricultural demand and sustainable development goals (Rosenzweig et al., 2001).

There is an urgent need to boost public investment in agriculture for GM technology to evolve in addressing the consequences of complex ecological dynamics between organisms and climate variability. However, some non-*Bt* cotton varieties offering superior results could also be part of the solutions provided to farmers (Gutierrez et al., 2020). For instance, studies have shown that adopting pure-line high-density short-season (non-*Bt* HD-SS) varieties of rainfed cotton could more than double current yields (Venugopalan et al., 2014; Kumar et al., 2020). It is also likely to avoid heavy pink bollworm infestations, thus reducing insecticides use. A more promising technology is the advanced molecular tools for precisely modifying plants using Clustered Regularly Interspaced Short Palindromic Repeats (CRISPR/Cas9) (Hu and Li, 2022). This tool allows the plant breeders to make targeted sequence variations, resulting in rapid crop improvements (Aziz et al., 2022).

## 5 Concluding remarks

Previous microlevel studies across developing countries show sizeable gains from *Bt* cotton in the initial years of its adoption. Yet, recent studies from India using aggregate data show modest benefits over extended periods. In this paper, I use new farm-level panel data from the Indian district of Ballari to show yield sensitivity to pest pressure has increased in the second decade of adoption, resulting in losses in some years. More specifically, despite *Bt* technology that is claimed to be protecting from pink bollworms, farmers suffered massive yield losses from the pest. It represents a significant threat to the livelihoods and the very lives of millions of subsistence Indian cotton farmers.

Unlike recent evidence using both aggregate- and farm-level data from China showing that *Bt* cotton remains economically beneficial in the short and long run, our findings from India show economic benefits can diminish in the long run. It raises an important question on the sustainability of *Bt* cotton, even with the second-generation *Bt* gene. One of the reasons for the recurrence of the pink bollworm in India currently debated is the non-compliance of farmers with refuge requirements. With short-run profitability leading to increased adoption of *Bt* cotton and decreased natural refuge crops, the long-run outcome can be disastrous. Thus, policymakers might need to address non-compliance urgently and, in countries without refuge policies, rethink mandating a non-*Bt* cotton refuge.

Even though Pakistan and China currently do not have a refuge policy, China successfully reversed low levels of pink bollworm resistance by planting second-generation hybrid seeds from crosses between *Bt* and non-*Bt* cotton, naturally increasing the refuge area with non-*Bt* plants randomly interspersed within fields of *Bt* cotton (Tabashnik and Carrière, 2019). Though the efficacy of the built-in natural refuge with seed mixture appears to be successful in China, this strategy for managing pest resistance in other countries remains to be experimented with.

Though this study provides evidence from one district in Karnataka, I suggest establishing independent studies with representative surveys across the cotton-growing states to determine the extent of returns from *Bt* cotton in light of the widespread pink bollworm infestation. Since *Bt* cotton is the only GM crop technology widely adopted by smallholder farmers, the findings can contribute to the broader public debate on the future of agricultural biotechnology in developing countries. This paper can inform the future scientific development of GM technology, which is expected to address food insecurity in the face of climate change.

## Data availability statement

The aggregate data used for the study and the STATA codes for the statistical analysis based on the regression model is freely available online at https://reshare.ukdataservice.ac.uk/853079/.

## Author contributions

The author confirms being the sole contributor of this work and has approved it for publication.
